# A Novel Naturally Occurring Class I 5-Enolpyruvylshikimate-3-Phosphate Synthase from *Janibacter* sp. Confers High Glyphosate Tolerance to Rice

**DOI:** 10.1038/srep19104

**Published:** 2016-01-12

**Authors:** Shu-yuan Yi, Ying Cui, Yan Zhao, Zi-duo Liu, Yong-jun Lin, Fei Zhou

**Affiliations:** 1 State Key Laboratory of Agricultural Microbiology, College of Life Science and Technology, Huazhong Agricultural University, Wuhan 430070, China; 2 National Key Laboratory of Crop Genetic Improvement and National Centre of Plant Gene Research, Huazhong Agricultural University, Wuhan 430070, China

## Abstract

As glyphosate is a broad spectrum herbicide extensively used in agriculture worldwide, identification of new *aroA* genes with high level of glyphosate tolerance is essential for the development and breeding of transgenic glyphosate-tolerant crops. In this study, an *aroA* gene was cloned from a *Janibacter* sp. strain isolated from marine sediment (designated as *aroA*_*J. sp*_). The purified aroA_*J. sp*_ enzyme has a *K*_*m*_ value of 30 μM for PEP and 83 μM for S3P, and a significantly higher *K*_*i*_ value for glyphosate (373 μM) than aroA_*E. coli*_. AroA_*J. sp*_ is characterized as a novel and naturally occurring class I aroA enzyme with glyphosate tolerance. Furthermore, we show that aroA_*J. sp*_ can be used as an effective selectable marker in both *japonica* and *indica* rice cultivar. Transgenic rice lines were tested by herbicide bioassay and it was confirmed that they could tolerate up to 3360 g/ha glyphosate, a dosage four-fold that of the recommended agricultural application level. To our knowledge, it is the first report of a naturally occurring novel class I *aroA* gene which can be efficiently utilized to study and develop transgenic glyphosate-tolerant crops, and can facilitate a more economical and simplified weed control system.

The shikimate pathway is essential for the biosynthesis of aromatic amino acids in plants, fungi and microorganisms^1^. The enzyme 5-enolpyruvylshikimate-3-phosphate synthase (EPSPS, EC2.5.1.19), also called aroA enzyme, plays a crucial role in the penultimate step of the shikimate pathway by catalyzing the transfer of the enolpyruvyl moiety of phosphoenolpyruvate (PEP) to the 5-hydroxyl group of shikimate-3-phosphate (S3P) to form 5-enolpyruvylshikimate-3-phosphate (EPSP). Glyphosate is the most widely used broad spectrum herbicide that mimics the carbocation state of PEP and binds EPSPS competitively^2^,^3^,^4^. The inhibition of EPSPS is a reversible reaction in which glyphosate binds to the binding site of PEP and forms a stable but non-covalent ternary complex with the enzyme and S3P (EPSPS-S3P-glyphosate)^5^. The ternary complex blocks the formation of EPSP, affecting the growth of the organism. Glyphosate provides an effective, efficient and economical way to control weeds^6^. As glyphosate is an unselective herbicide, the development of glyphosate-tolerant crops has dramatically changed weed management practices and increased the yield of crops.

Since the mutant *aroA* gene cloned from *Salmonella typhimurium* was first reported in 1983^7^, the aroA enzyme (EPSPS) identified as the target of glyphosate resistance has been studied extensively over the past three decades^8^. Different aroA enzymes from various organisms have been divided into two classes on the basis of their intrinsic glyphosate sensitivities and their substantial sequence variations^9^. In general, class I aroA enzymes are naturally sensitive to glyphosate and generally found in *Escherichia coli*, *Aeromonas salmoncida*, *Petunia hybrida* and *Arabidopsis thaliana*. Class II aroA enzymes share less than 30% sequence similarity with class I enzymes, and can retain their activity at a high concentration of glyphosate, which are isolated from *Agrobacterium tumefaciens* CP4, *Pseudomonas* sp. strain PG2982, *Bacillus subtilis*, *Ochrobactrum anthropi*, *Staphylococcus aureus* and other bacteria species^10^,^11^,^12^,^13^,^14^,^15^,^16^. In class II enzymes, two conserved regions have been proved to be key regions involved in glyphosate tolerance: RXHXE and NXTR (X represents non-conserved amino acids), in both of which the positive charge of the side chain of Arg hinders the binding of glyphosate^17^.

Several class II enzymes have been used to generate transgenetic plants^18^,^19^,^20^,^21^,^22^. Among them, only the one from *Agrobacterium tumefaciens* CP4 has been used for the production of transgenic glyphosate-tolerant crops.

Glyphosate insensitivity can be also achieved in class I aroA enzymes through site-directed mutagenesis^23^,^24^,^25^ or natural selection^26^,^27^,^28^. However, most of the mutants show lower affinity for their substrate PEP. Among them, the double mutated aroA (TIPS) has been successfully used to produce the first commercial transgenic glyphosate-tolerant maize (GA21). In recent years, considerable attention has been paid to the exploration of new types of aroA enzymes with commercial feasibility.

In this study, a novel aroA enzyme from a glyphosate-tolerant strain *Janibacter* sp (designated as aroA_*J. sp*_) was isolated from marine sediment and further functionally characterized. Through the sequence analyses and phylogenetic analyses, aroA_*J. sp*_ is characterized as a new class I aroA enzyme. The *K*_*m*_ values for PEP and S3P of this enzyme were found to be similar to those of the analog from aroA_*E. coli*_, but its *K*_*i*_ value for glyphosate was significantly higher. Finally, the function of aroA_*J. sp*_ was evaluated in both *Japonica* and *Indica* rice cultivar, zhonghua11 (ZH11) and minghui86 (MH86), respectively. The results show that it can be used as an effective marker for direct selection and the generated transgenic rice lines were conferred high glyphosate tolerance (up to 3360 g/ha glyphosate). Taken together, our study indicates that the naturally occurring novel class I *aroA*_*J. sp*_ gene has promising potential for the development and breeding of transgenic glyphosate-tolerant crops.

## Results

### Isolation of gene conferring glyphosate tolerance

With the aim of finding novel *aroA* genes, the marine sediment sample was enriched with glyphosate and plated on M9 agar plates containing different concentrations of glyphosate. After plate-screening, one strain (named as L42) grew very well at a concentration of 150 mM glyphosate. The L42 strain was gram-positive and coccoid, whose 16S rRNA sequence (1487 bp) displayed the highest similarity (99%) with that of *Janibacter.*sp *N2M.* Thus, this strain was identified as a *Janibacter* species. To isolate the gene encoding aroA enzyme from L42 strain, a genomic library was constructed. After screening about 5,000 colonies from the library, one positive colony was found to possess the ability to grow on M9 agar containing 100 mM glyphosate, indicating that the recombinant plasmid in the colony might contain a gene involved in glyphosate tolerance. From this colony the plasmid (named as pZY3) was recovered.

### Sequence analysis of *aroA*_*J*. *sp*_ gene

DNA sequencing analysis revealed that the pZY3 plasmid contained a 1,299 bp DNA fragment that consisted of a complete open reading frame (ORF1) and encoded 432 amino acids with an estimated molecular weight of 45.1 kDa. The overall GC content of the ORF1 was 73.6%. The deduced amino acid sequence was then used to search for homologous sequences through the BLAST program, and the protein was found to share the highest homology (67% amino acid identity) with aroA enzyme from *Janibacter* sp HTCC2649 (GenBank: EAP99947.1), indicating that the ORF1 from L42 strain encoded an aroA enzyme. Thus, the *aroA* gene from the *Janibacter.sp* was designated as *aroA*_*J. sp*_.

**Figure 1:**
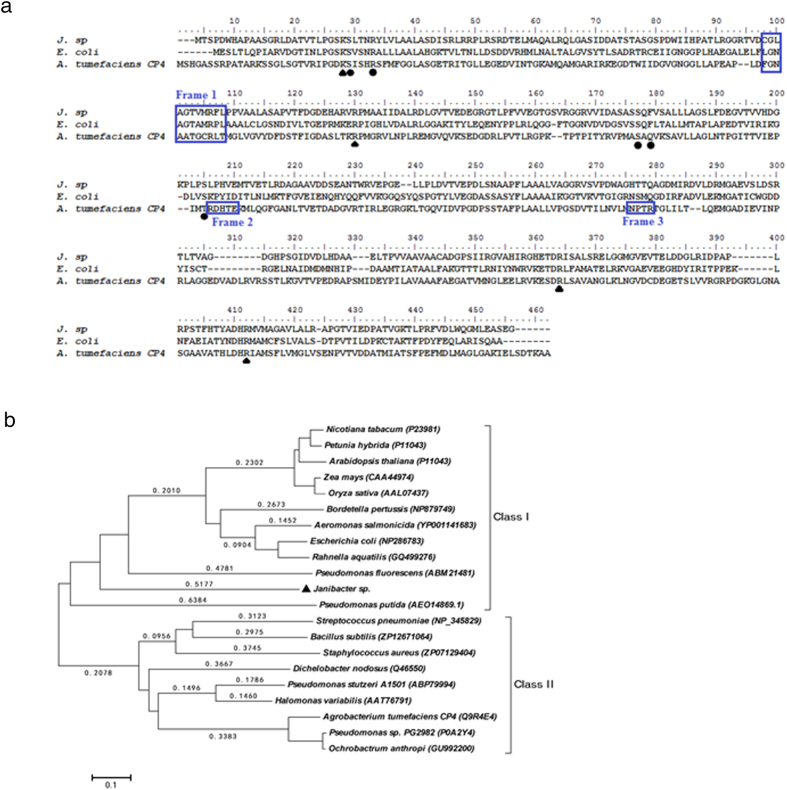
(**a**) Multiple alignments of amino acid sequences from aroA_*J. sp*_ with representative class I and class II aroA enzymes using ClustalW program. Triangles, residues critical for PEP binding; and circles, residues critical for S3P binding. The two regions involved in glyphosate resistance in class II aroA enzymes are boxed in Frames 2 and 3. The motif important for interaction with PEP conserved in class I aroA enzymes is boxed in Frame 1. (**b**) The phylogenetic tree was based on homologous sequences of the aroA proteins and the neighbor-joining methods (MEGA4.0). The percentage of the tree from 1000 bootstrap resamples supporting the topology is indicated when above 50. Accession numbers or international patent publication numbers are shown in parentheses. The scale bar represents 0.1 substitutions per position.

Multiple alignments of amino acids showed that aroA_*J. sp*_ shared only 26–35% amino acid identity with most class I and class II aroA enzymes. Several key residues involved in S3P and PEP binding were well conserved in aroA_*J. sp*_, including S24, K25, S26, R127, S173, Q175, R348, R389, S202, R348 and R389, which are indicated in two classes of the aroA enzymes in Fig. 1. The highly conserved region containing residues XLGNAGTAXRXL (Fig. 1a, Frame 1) has been demonstrated to be critical for the substrate PEP binding in aroA enzymes^26^. It was indeed present in aroA_*J. sp*_, and showed higher sequence identity with that in class I aroA enzymes than that in class II analogs. In addition, two other regions RXHTE (Fig. 1a, Frame 2) and NPTR (Fig. 1a, Frame 3), which are well conserved in the class II aroA enzymes, were completely absent in aroA_*J. sp*_. Further phylogenetic analysis indicated that aroA_*J. sp*_ belonged to class I aroA but showed little sequence similarity with other class I aroA enzymes (Fig. 1b). Taken together, these results suggest that aroA_*J. sp*_ is a new member of class I aroA enzymes based on its amino acid sequence.

### Glyphosate insensitivity assay of aroA_*J. sp*_ in *E. coli*

**Figure 2:**
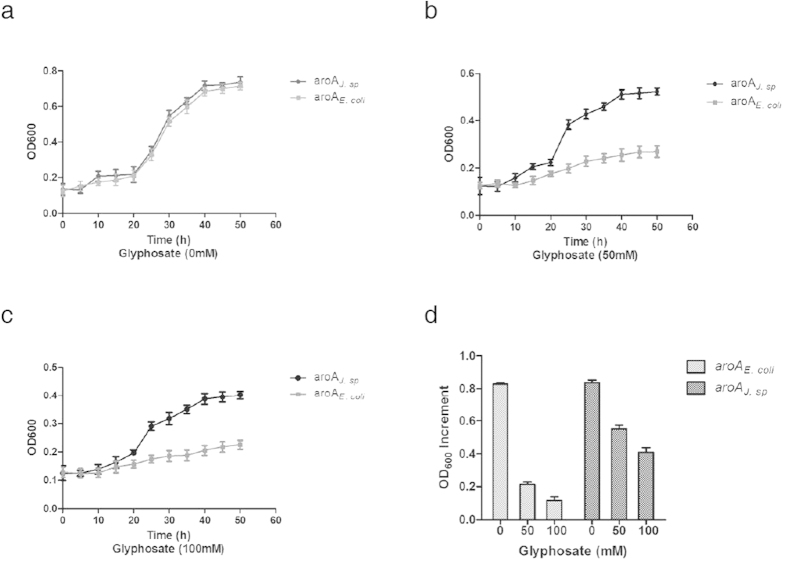
Growth curve of *E. coli* AB2829 harboring either pGEX-6p-1-*aroA*_*J. sp*_ or pGEX-6p-1-*aroA*_*E. coli*_ in liquid M9 minimal medium supplemented with glyphosate at concentrations of 0 mM (**a**) 50 mM (**b**) and 100 mM (**c**). (**d**) Bar charts of growth analysis of the strains. The results presented are the averages of two sets of experiments done in triplicate.

*E.coli* AB2829, which is deficient in *aroA*, can only grow in minimal medium when foreign *aroA* complements the deficiency. Plasmids pGEX-6p-1-*aroA*_*J. sp*_ and pGEX-6p-1-*aroA*_*E. coli*_ were obtained by cloning *aroA*_*J. sp*_ and *aroA*_*E. coli*_ into GST carrier vector pGEX-6p-1. Then, the function of the isolated *aroA*_*J. sp*_ gene in *E. coli* was investigated by comparing the growth characteristics of *E. coli* AB2829 harboring either plasmid pGEX-6p-1-*aroA*_*J. sp*_ or pGEX-6p-1-*aroA*_*E. coli*_ at glyphosate concentrations of 0, 50 and 100 mM. All the strains grew well in M9 medium without glyphosate, and the growth of each strain was about 80%, suggesting that *aroA*_*J. sp*_ gene has no adverse effect on the bacterial growth under this condition (Fig. 2a). However, bacterial cells containing *aroA*_*E. coli*_ were clearly inhibited in the presence of 50 mM glyphosate with the growth decreasing to 30% (Fig. 2b), and the cells were severely inhibited in the presence of 100 mM of glyphosate with the growth of only 20% (Fig. 2c). In contrast, cells carrying *aroA*_*J. sp*_ grew well under all the conditions with 50 or 100 mM of glyphosate. In medium containing 50 mM and 100 mM glyphosate, the growth of this strain was about 55% and 40%, which was 2.7 and 2-fold higher than that of the strain containing *aroA*_*E. coli*_ (Fig. 2d). These results indicate that *aroA*_*J. sp*_ can functionally complement the deficiency of *aroA* in *E. coli* AB2829 and definitely carries a high glyphosate-tolerant capability, whereas the *aroA*_*E. coli*_ gene derived from bacterium strain DH5α does not have such capability.

### Expression and purification of aroA_*J. sp*_ enzyme

After induction by IPTG, soluble expression of the aroA_*J. sp*_-GST fusion was performed in bacterium strain BL21, and the induced fusion protein, which comprised an aroA_*J. sp*_ (47 kDa) and a GST tag (26 kDa), showed an expected molecular mass of 73 kDa on SDS-PAGE (Fig. S2a). Affinity-purified aroA_*J. sp*_ -GST fusion was cleaved by 3C protease, resulting in a 47 kDa aroA_*J. sp*_ protein with high purity as indicated by a single band on SDS-PAGE. Soluble protein was successfully produced in BL21 under the conditions used in this study, suggesting a proper folding and conformation for its functionality. GST carrier within the aroA_*J. sp*_ -GST fusion may play a role for this soluble expression. At the same time, the aroA_*E. coli*_-GST fusion was expressed in parallel and run on the same SDS-PAGE as a control, and aroA_*E. coli*_ of a similar size was also obtained after cleavage of the fusion (data not shown). The concentration of the purified aroA_*J. sp*_ and aroA_*E. coli*_ enzyme was 0.3 mg/ml and 1.5 mg/ml, respectively.

### Kinetic properties of purified aroA_*J. sp*_ enzyme

As shown in Fig. S2b and S2c, aroA_*J. sp*_ enzyme had the highest activity at pH 8.0 and 40 °C, and maintained about 40% activity over a pH range from 6 to 10 and 30% activity over a temperature range from 10 to 60 °C, implying high stability and applicability of this enzyme under different conditions.

**Figure 3:**
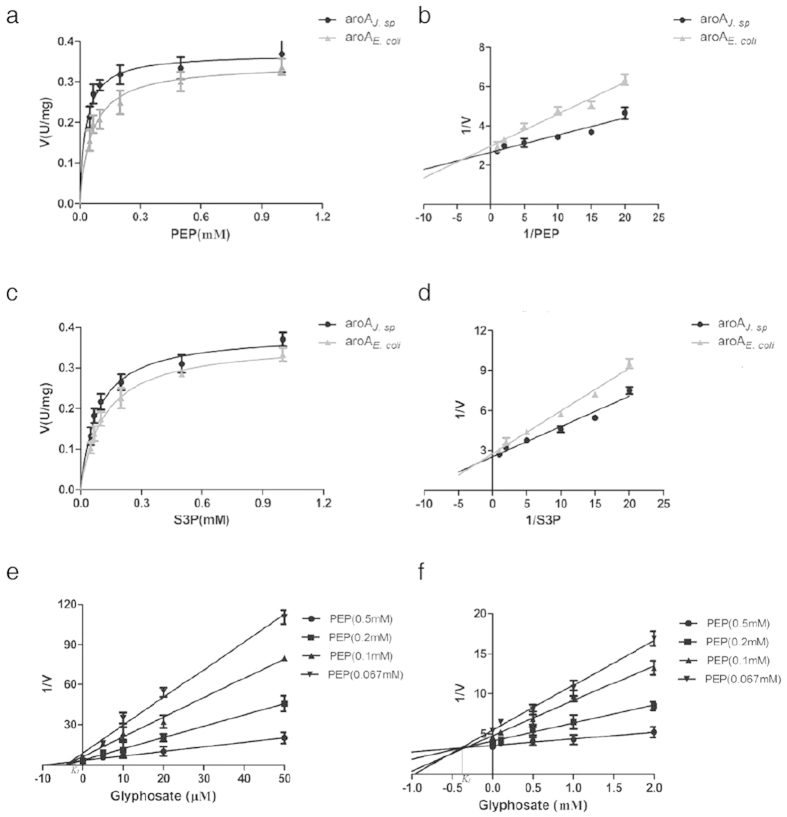
(**a**) The V-S curve of aroA_*J. sp*_ and aroA_*E. coli*_ assayed at fixed S3P and various PEP concentrations. (**b**) The Lineweaver-Burk plots aroA_*J. sp*_ and aroA_*E. coli*_ assayed at fixed S3P and various PEP concentrations. (**c**) The V-S curve of aroA_*J. sp*_ and aroA_*E. coli*_ assayed at fixed PEP and various S3P concentrations. (**d**) The Lineweaver-Burk plots aroA_*J. sp*_ and aroA_*E. coli*_ assayed at fixed PEP and various S3P concentrations. (**e**) The *K*_*i*_ values of aroA_*E. coli*_ determined by lines converging on the x-axis of Lineweaver-Burk plots. (**f**) The *K*_*i*_ values of aroA_*J. sp*_ determined by lines converging on the x-axis of Lineweaver-Burk plots.

The proteins encoded by aroA_*J. sp*_ and aroA_*E. coli*_ were purified separately with a GST system and used for enzymatic activity assay. Due to the structural difference from class II enzymes, class I aroA enzymes are known to be naturally glyphosate-sensitive. The *K*_*m*_ value for PEP and the *K*_*i*_ value for glyphosate of the purified aroA_*E. coli*_ enzyme were determined to be 60 and 0.9 μM in this experiment, respectively, and the corresponding *K*_*i*_/*K*_*m*_ ratio was 0.015. Kinetic constants of the aroA_*J. sp*_ enzyme were significantly different from those of the aroA_*E. coli*_ enzyme (Table 1, Fig. 3). In the presence of KCl, The *K*_*m*_values of the aroA_*J. sp*_ enzyme for PEP and S3P were 30 and 83 μM, respectively, which were lower than those of aroA_*E. coli*_, indicating a high substrate affinity of the aroA_*J. sp*_ enzyme. The IC_50_ and the *K*_*i*_ values for glyphosate of the aroA_*J. sp*_ enzyme were 3.6 mM and 373 μM, respectively, and the IC_50_ value was 90-fold that of the aroA_*E. coli*_ enzyme. The *K*_*i*_/*K*_*m*_ value of aroA_*J. sp*_ enzyme was calculated to be 12.4, which is 800-fold that of aroA_*E. coli*_, suggesting that the aroA_*J. sp*_ enzyme has a higher level of glyphosate tolerance than the aroA_*E. coli*_ counterpart.

### Nuclear transformation directly using *aroA*_*J. sp.*_ as selectable marker

**Figure 4:**
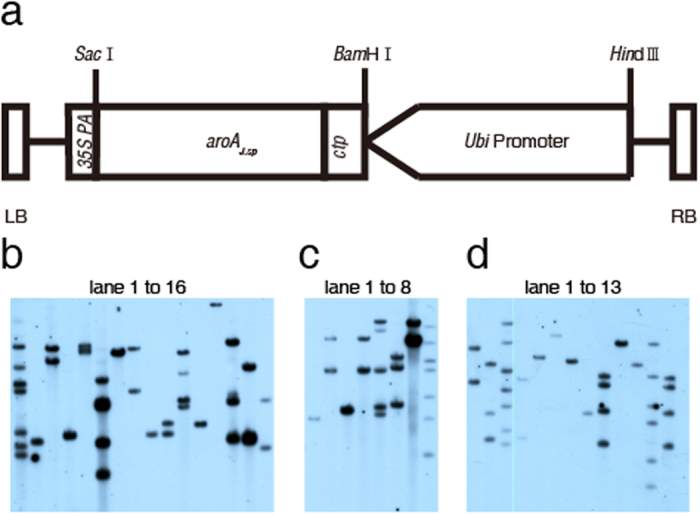
(**a**) the expression cassette of pU130-aroA_*J. sp*_. The *ctp-aroA*_*J. sp*_ fusion gene was controlled by a maize ubiquitin promoter and *35S PolyA* terminator. (**b–d**) Southern blot analysis of positive transgenic T_0_ *Japonica* plants of cv. ZH11 from 3 independent transformations. (**b**) lane 1–16, (**c**) lane 1–8 and (**d**) lane 1–13 represent different transgenic events.

Since the aroA_*J. sp*_ showed high glyphosate-tolerant capability in *E.coli* and had a high *K*_*i*_/*K*_*m*_ value, it was tested in a *japonica* rice variety zhonghui11 (ZH11) to explore the possibility of employing *aroA*_*J. sp*_ as a selectable marker in crops. To ensure efficient expression of *aroA*_*J. sp*_, maize Ubi promoter was used, because it conferred a higher level of foreign gene expression than Act1 and CaMV 35S promoters in monocot study^29^. Since aroA was located in the chloroplast in plants, a chloroplast transit peptide from *Arabidopis thaliana* (CTP) was added to the N-terminus of aroA_*J. sp*_ to target the protein into the chloroplasts in rice. The resulted pU130-*aroA*_*J. sp*_ (Fig. 4a) was transformed into ZH11.

From 3 independent transformation experiments, glyphosate-resistant calli were selected on medium containing 200 mg/L glyphosate. Resistant calli appeared after 5 weeks on average and could be transferred to the differentiation medium after one more week, resulting in a callus formation rate of around 50% (Table 2).

16, 8 and 13 resistant lines were obtained out of 84, 67 and 75 resistant calli, respectively. All T_0_ lines were confirmed by PCR (data not shown) and Southern blot analysis (Fig. 4b–d). The results tentatively suggest that direct selection for glyphosate tolerance using *aroA*_*J. sp*_ as selectable marker can readily produce transgenic rice lines with a high transgenic positive rate.

### Glyphosate tolerance of the transgenic *indica* rice

**Figure 5:**
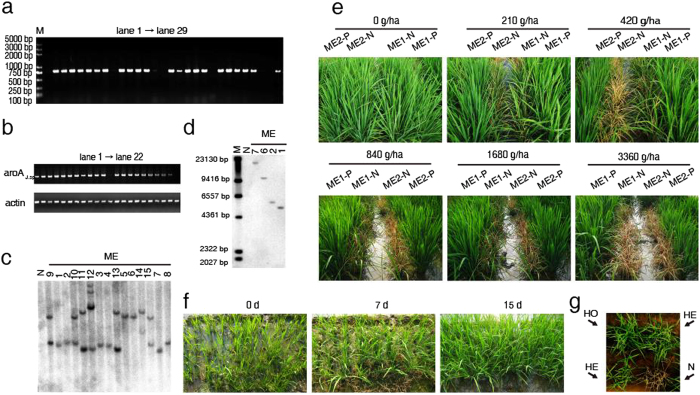
(**a**) PCR analysis of transgenic T_0_ *indica* generation plants. M, Trans2K Plus DNA Marker; lane 1, untransformed MH86; lane 2 to 29, represents transgenic events P1-P28. (**b**) expression of *aroA*_*J. sp*_ cDNA in transgenic rice plants. *Upper*, Lanes 1 to 22, represents transgenic plants PP1-PP22, N, the non-transgenic rice control plant. *Nether*, the *actin* gene amplified from all the tested samples. (**c**) Southern blot analysis of transgenic T_0_ *indica* plants of cv. MH86 with *Hin*d III digestion. N, untransformed MH86; lane 1–15, different transgenic lines, corresponding sample numbers were marked. (**d**) Southern blot analysis of transgenic T_2_ *indica* generation plants with *Sac*I digestion. Lane 1, untrasformed MH86; lane 2–5, confirmed single copy, corresponding sample numbers were marked. (**e**) glyphosate tolernace of T_2_ transgenic rice plants under different glyphosate dosages. T_2_ rice seeds were germinated in field and grown to tillering stage. 0 g/ha, 210 g/ha, 420 g/ha, 840 g/ha, 1680 g/ha and 3360 g/ha glyphosate (the corresponding applied concentration of glyphosate to each dosage was 0 mg/L, 187.5 mg/L, 375 mg/L, 750 mg/L, 1500 mg/L and 3000 mg/L) were sprayed on ME1 and ME2 transgenic lines along with non-transformed control plant and photograph was taken 1 week after spray. ME1-P and ME2-P represent the homozygous transgenic lines of ME1 and ME2, while ME1-N and ME2-N are their corresponding negative transgenic lines. (**f**) glyphosate tolerance test of T_4_ generation seedlings of ME1 line. The 28-day-old ME1 T_4_ generation seedlings were transplanted in the field with weeds and sprayed with 840 g/ha glyphosate (the corresponding applied concentration of glyphosate was 840 mg/L). Photographs were taken by Y.C. at day 0, 7 and 15 after spray. (**g**) Selection of homozygous line by glyphosate. Homozyous (HO), heterzygous (HE) T_2_ transgenic rice plants along with untransformed control plants (N) were sprayed with 1% (vol/vol) solution of the herbicide Roundup containing 41% isopropylamine salt of glyphosate, and photograph was taken by Y.C. at 3 d after spray.

As *aroA*_*J. sp*_ could be used as direct selectable marker in *japonica* rice, we further applied it in a conventional *indica* rice variety, minghui86 (MH86). After 8 weeks of selection followed by the differentiation of calli, 28 putative transgenic plants were generated (named as P1-P28). The transformation efficiency for MH86 was about 5% based on the numbers of the total transformants and the calli used for transformation. PCR analysis showed that a specific fragment of 815 bp was amplified from 22 of the 28 T_0_ transgenic lines, indicating an integration of *aroA*_*J. sp*_ gene into the rice genome (Fig. 5a). Those PCR positive lines were then named as PP1-PP22, and chosen for RT-PCR analysis (Fig. 5b). The result demonstrated a proper transcription of the integrated *aroA*_*J. sp*_ gene in 20 rice transformants (marked as ME1-20). The copy number of T-DNA integration in these T_0_ transgenic lines (ME1-20) was determined by Southern blotting (Fig. 5c). T_2_ progenies from four lines (ME1, ME2, ME6 and ME7) were further tested by Southern blotting. The result confirmed that in these lines, the transgene was integrated into the genome as a single copy and was stably inherited (Fig. 5d). In order to further assay the tolerant capability for crop breeding, the ME1 line and ME2 line were chosen for further characterization, because their T-DNA insertions were mapped to the intergenic region (data not shown). The data collected from the field experiment for agronomic performance showed the homozygous T_2_ transgenic rice lines (ME1-P and ME2-P) had no significant differences from the their corresponding negative transgenic lines (ME1-N and ME2-N) (Table 3).

Spray with glyphosate resulted in a strong inhibition of growth and the ultimate death of the non-transgenic rice plants (210 g/ha), but did not influence the normal growth of the homozygous T_2_ transgenic rice plants at 3360 g/ha glyphosate (Fig. 5e).

In addition, T_4_ generation seedlings of ME1 line were transplanted into a weedy field without weed control. Only 7 days after spray with 840 g/ha glyphosate, the weeds were dead, while the transgenic rice plants reached to tillering stage, and grew normally 15 days after spray (Fig. 5f), suggesting the applicable potential of *aroA*_*J. sp*_ in weed control.

## Discussion

In modern agricultural system, herbicides have greatly contributed to the reliable global food production as they can easily remove weeds. Among these chemicals, glyphosate is the most widely used one due to its broad-spectrum herbicidal activity and minimal human and environment toxicity. Since the glyphosate use pattern commenced in 1996, the planting area of transgenic herbicide-tolerant crops accounts for more than 80% of the total planting area of transgenic crops in 2014 (ISAAA Annual Report: 2014 http://www.isaaa.org/resources/publications/annualreport/2014/pdf/ISAAA-Aannual_ Report-2014.pdf). In the last three decades, a number of promising enzymes were identified. However, among those enzymes, only two aroA variants have been utilized for developing commercial glyphosate-tolerant crops: CP4, a naturally occurring class II type, and TIPS, a mutant class I type. Therefore, identification of more novel glyphosate-insensitive *aroA* genes which can be used to generate glyphosate-tolerant crops with commercial feasibility will bring great positive impact on global food security by facilitating a more economical and simplified weed control system.

Mutagenesis is one method to improve the glyphosate tolerance of aroA enzymes, but when the tolerance for glyphosate is increased, the binding affinity for PEP and catalytic efficiency may be decreased at the same time, as glyphosate and PEP bind to the same site. To date, TIPS is the only class I enzyme which is essentially insensitive to glyphosate but maintains high affinity for PEP. Thus, the exploration of new types of aroA enzymes with both intrinsic glyphosate tolerance and high affinity with PEP is very necessary.

Microbial biodiversity provides opportunities to extract genes and proteins with unique properties for industrial and environmental applications. As a result, in this study, a *Janibacter* sp strain that could grow at a 150 mM concentration of glyphosate was isolated from marine sediment, and the *aroA* gene from this strain was cloned and named as *aroA*_*J. sp*_. Phylogenetic analysis and conserved domain analysis revealed that aroA_*J. sp*_ is characterized as an novel and naturally occurring class I aroA enzyme but with glyphosate tolerance.

*K*_*i*_/*K*_*m*_ of aroA enzyme is often seen as an important indicator of enzymatic activity in the presence of glyphosate. The overexpression of class I type AM79 aroA enzyme, whose *K*_*i*_/*K*_*m*_ value is 10.6, resulted in high glyphosate tolerance in tobacco^27^. The double mutated maize aroA (TIPS), which also belongs to class I type aroA enzyme, has also relatively high *K*_*i*_/*K*_*m*_ ratio (5.8)^30^. According to ISSSA GM approval database, *TIPS* has been used for generating glyphosate-tolerant crops, resulting in 38 events (http://www.isaaa.org/gmapprovaldatabase/default.asp). The *K*_*i*_/*K*_*m*_ value of aroA_*J. sp*_ enzyme was calculated to be 12.4 (Table 1), suggesting that aroA_*J. sp*_ maintains a high affinity with PEP and intrinsic glyphosate tolerance.

The mechanism of resistance to glyphosate of class I type has been extensively investigated. Take *aroA*_*E.coli*_ for example: G96A substitution was shown to increase the glyphosate tolerance to 100 folds, but decrease the binding affinity for PEP. Interestingly, the corresponding site in CP4 gene (class II type) is also an A. We attempted to conduct G96A substitution. Surprisingly, it turned out that the enzyme activity dramatically decreased to be almost inactive (data not shown). It was also reported that the P106L mutant of rice (*Oryza sativa*) aroA enzyme has a high glyphosate resistance while retaining relatively high catalytic efficiency^31^. Besides, it was claimed that substituting T97 with I or L and P101 with T, G, C, A or I could ensure both intrinsic glyphosate tolerance and high affinity with PEP^30^. P101 substitution confers relatively low glyphosate tolerance while maintains high catalytic efficiency, but only the mutation of both T97 and P101 can provide the conformational changes and lead to high catalytic efficiency and glyphosate tolerance. For our newly identified aroA_*J. sp*_, the corresponding amino acids are T and F, which get around patent protection. Therefore, this newly identified aroA_*J. sp*_ might provide a new aspect to reveal the function mechanism of class I type aroA enzymes. And further mutagenesis work is needed to further improve the glyphosate tolerance of this enzyme.

Selectable marker gene is essential in the production of transgenic plants. Most of the marker genes are usually either antibiotic or herbicide resistant genes^32^, such as *hpt* (hygromycin phosphotransferase gene), *nptII* (neomycin phosphotransferase gene), and *bar* (phosphinothricin acetyltransferase gene)^33^. Although glyphosate tolerant gene *aroA* has been used directly as selectable marker gene in *Arabidopsis* and other species^34^,^35^,^36^, there are few reports of its application in rice. In most of the reported glyphosate-tolerance rice events, *hpt* was used as the selectable marker gene^22^,^37^. Zhao *et al.* used a glyphosate-tolerant *aroA* variant *G6 gene* as the selectable marker gene in a *japonica* rice XiuShui-110^21^. Although several transgenic lines were generated, the PCR positive rate was rather low, and the Southern blotting results indicated that most of the lines were multiple-copy integration lines. In our study, the transformed calli from both *japonica* and *indica* rice were selected on medium containing 200 mg/L glyphosate, and transgenic rice lines were readily obtained (Table 2 and data not shown). Therefore, it can be speculated that *aroA*_*J. sp*_ can be directly used as a selectable marker gene for its high glyphosate tolerance capability.

Rice can be significantly influenced by glyphosate, and the improper use of glyphosate can cause severe injury to rice and reduce grain yield^38^. The planting area of transgenic herbicide-tolerant crops accounts for more than 80% of the total planting area of transgenic crops in 2014 (ISAAA Annual Report: 2014 http://www.isaaa.org/resources/publications/annualreport/2014/pdf/ISAAA-Aannual_ Report-2014.pdf). However, when other glyphosate-tolerant crops have been adopted^9^,^25^, no glyphosate-tolerant rice varieties have been commercialized. In our study, the untransformed MH86 was sensitive to glyphosate, and were dead 7 days after 420 g/ha glyphosate treatment; while transgenic MH86 rice plants expressing *aroA*_*J. sp*_ exhibited robust tolerance to glyphosate at a dosage of 3360 g/ha, which is four-fold that of the recommended dosage (Fig. 5e) The tolerant level was comparable with that resulted from the expression of several class II *aroA* genes in other plants^20^,^22^,^39^.

At the early stage of transplanting, the rice growth is often affected by weeds. Here, our results show that the spray of the recommended amount of glyphosate (840 g/ha) can efficiently control the weeds and has no apparent effect on the growth of *aroA* transgenic lines (Fig. 5f). The field assay indicated that glyphosate-tolerant line ME1 can be powerful in fighting against weeds, and thus can be used in no-till or conventional till system. In addtion, we observed that the homozygous and heterzygous T_2_ lines can be easily distinguished from nontransformant lines with the spray of 1% (vol/vol) solution of the herbicide Roundup which contains 41% isopropylamine salt of glyphosate (Fig. 5g). MH86 has been used as an importnat conventional restored line in hybrid seed production. When the *aroA*_*J. sp*_ MH86 rice line is used as the paternal line, its next generation will keep the glyphosate tolerance ability. This characteristic facilitates the identification of false hybrids, which helps to improve the qutlity of hybrids and to ensure the sustained development of food production.

In conclusion, *aroA*_*J. sp*_ is characterized as a new type of class I aroA enzyme with glyphosate tolerance, which provides new insights into the functions of class I aroA enzymes. We believe that *aroA*_*J. sp*_ gene has a promising potential for the development and breeding of transgenic glyphosate-tolerant crops, which will facilitate a more economical, complete and simplified weed control.

## Methods

### Materials and Mediums

Marine sediment was sampled at site IR-CTD5(16°59.9412′N 124°58.2958′E) on the south west Indian Ridge. The glyphosate tolerant bacteria *Janibacter* sp was isolated from the marine sediment. Bacterial strains, *Escherichia coli* DH5α, *E. coli* aroA mutant strain AB2829, *E. coli* BL21 (DE3) and the zhonghua11 *japonica* rice variety were stored in our laboratory. The pUC118 vector was purchased from Takara (Japan), and the vector pGEX-6p-1 was kept in our laboratory. Vector pCAMBIA1300 was a gift from the Center of the Application of Molecular Biology to International Agriculture, Australia. All enzymes used for restriction digestion and ligations were purchased from Takara (Japan). S3P was purchased from Santa Cruz Biotechnology (Santa Cruz, CA, USA). PEP and glyphosate were purchased from Sigma (St. Louis, MO, USA). All chemicals were of analytical grade. The medium used for isolating glyphosate tolerant strain was minimal medium M9 (6.8 g/L Na_2_HPO_4_, 3.0 g/L K_2_HPO_4_, 1.0 g/L NH_4_Cl, 0.5 g/L NaCl and 0.12 g/L MgSO_4_) prepared in artificial sea water (distilled water with 0.5 g/L MgCl_2_, 0.5 g/L MgSO_4_, 0.5 g/L CaCl_2_, 0.55 g/L KCl, 0.16 g/L NaHCO_3_, 0.08 g/L KBr, 34 mg/L SrCl, 22 mg/L H_3_BO_3_, 4 mg/L Na_2_SiO_3_, 2.4 mg/L NaF), and the minimal medium used for the glyphosate tolerance test in *E. coli* was M9 (6.8 g/L Na_2_HPO_4_, 3.0 g/L K_2_HPO_4_, 1.0 g/L NH_4_Cl, 0.5 g/L NaCl and 0.12 g/L MgSO_4_) supplemented with 0.4% (w/v) glucose as a carbon source plus the appropriate antibiotics.

### Isolation of glyphosate-tolerant strain

The marine sediment was used for enrichment with glyphosate for 7 d, and then the enriched culture was diluted with distilled water and plated on M9 agar plates containing 60 mM glyphosate. After 48 h of incubation at 28 °C, the colonies were further screened at 100 and 150 mM glyphosate concentrations. One strain numbered L42 grew well in the presence of 150 mM glyphosate and was chosen for further study. The 16S rRNA sequence of L42 strain was used for species identification.

### Isolation of gene associated with glyphosate tolerance

A genomic library of the L42 strain was constructed. The chromosomal DNA was extracted from the strain and partially digested with *Sau3*AI to produce 4–9 kb fragments. These DNA fragments were inserted into the pUC118 vector^40^. The ligation mixture was incubated at 4 °C overnight and transformed into *E. coli* DH5α. The transformants containing the recombinant pUC118 were transferred onto M9 agar plates containing 20 mM glyphosate and then the plates were incubated at 37 °C for 48 h. One colony exhibiting glyphosate tolerance was isolated from the plates containing a recombinant plasmid (pZY3) with an insert of approximately 3.5 kb.

### Sequence analysis

The inserted fragment from the plasmid pZY3 was sequenced by the Genescript Company (Nanjing, China), and the nucleotide sequences were analyzed using the Softberry Gene Finding Tool (http://linux1.softberry.com/berry). Sequence alignments were performed using the BLAST program (http://blast.ncbi.nlm.gov/blast) combined with the ClustalW program software^41^. The phylogenetic tree of aroA_J. sp_ was constructed by MEGA5^42^.

### Construction of prokaryotic expression vectors pGEX-6p-1-*aroA*_*J. sp*_ and pGEX-6p-1-*aroA*_*E. coli*_

The oligonucleotide primer sequences were as follows: primer 1 (5′-CGCGGATCC ATGACCAGTCCTGATTGGCATGC-3′) (the *Bam*HI site is underlined); primer 2 (5′-CCGGAATTCTCAGCCCTCCGACGCCTCG-3′) (the *Eco*RI site is underlined), which was designed according to the sequence derived from the insert of plasmid pZY3 and supplied by the Genescript; primer 3 (5′-CGCGGATCCATGGAA TCCCTGACGTTACAACCCATCGCTCGTG-3′); and primer 4 (5′-CCGGAATTC TCAGGCTGCCTGGCTAATCCGCGCCAGCT-3′), which was specific for amplifying *aroA*_*E. coli*_ gene and was designed based on the sequence available in GenBank (GenBank:X00557). The *aroA*_*J. sp*_ and *aroA*_*E. coli*_ genes were amplified, using the recombinant plasmid pZY3 and the genomic DNA from *E. coli* as the template, respectively. The *FastPfu* DNA polymerase (Transgen, Beijing, China) was used for all the reactions under the following conditions: 30 cycles of 94 °Cfor 20 sec, 55 °C for 20 sec, and 72 °C for 1 min. After PCR, the amplified products were gel-purified, digested with *Bam*HI and *Eco*RI, and ligated into the pGEX-6p-1 vector.

### Cell growth in the presence of glyphosate

Strain *E. coli* AB2829^31^ was transformed with pGEX-6p-*aroA*_*J. sp*_ plasmid and the transformants were cultured in 50 mL LB medium containing 100 μg/mL ampicillin at 37 °C until the OD_600_ values reached 0.1. The the culture was collected by centrifugation and washed twice using liquid M9 minimal medium. Bacteria in the culture were then grown with shaking at 37 °C in liquid M9 minimal medium supplemented with glyphosate at the concentrations of 0, 50 and 100 mM. The OD_600_ values of the cultures were determined at approximately 5-h intervals to record the growth rates of the strains till 50 h. OD_600_ increments were calculated as (OD_a_-OD_b_)/OD_0_, in which OD_a_ and OD_b_ represent the OD_600_ value before and after growing for 50 h, and OD_0_ is the final OD_600_ value without glyphosate. The results presented are the averages of two sets of experiments done in triplicate. The strain AB2829 harboring pGEX-6p-*aroA*_*E. coli*_ was used as the control.

### Expression and purification of aroA_*J. sp*_ and aroA_*E. coli*_ enzymes

*E. coli* BL21 (DE3) was transformed separately with pGEX-6p-1-*aroA*_*J. sp.*_ and pGEX-6p-1-*aroA*_*E. coli*_ plasmids, and cultivated in LB medium containing 100 μg/mL ampicillin at 37 °C until the OD_600_ values reached 0.6–1.0. IPTG (0.5 mM) was then added, and the cultures were further incubated at 18 °C for 8 h. The cells were harvested and resuspended in 50 ml Hepes buffer (50 mM Hepes, 100 mM KCl, and 2 mM dithiothreitol, pH 7.0). After treatment by high pressure crushing, the cells were centrifuged at 10,000 × g for 40 min at 4 °C, and the supernatant was loaded onto a Glutathione-S-Transferase (GST) agarose at 4 °C. The glutathione-S-transferase (GST)-tagged aroA enzymes were purified using a GST Fusion System (GE Healthcare Sweden). The GST tag was removed by digestion with a 3C protease solution (10 U/μl, PreScission; Pharmacia). The molecular mass of the protein was determined by a 12% sodium dodecyl sulfate polyacrylamide gel electrophoresis (SDS-PAGE), and the enzyme concentration was measured by a Bradford Protein assay kit (Sangon, China).

### Enzyme assay

AroA enzyme activity was determined by measuring the release of inorganic phosphate using the malachite green dye assay method^43^. The reaction (final volume, 50 μL) was performed at 28 °C in 50 mM Hepes buffer (pH 7.0), 1 mM S3P, 1 mM PEP, and 0.4 μg of the purified enzyme. After incubation for 4 min, 0.8 mL malachite green (0.045%) /ammonium molybdate (4.2%) colorimetric solution was added; 1 min later, the reaction was terminated by adding 0.1 mL 34% (w/v) sodium citrate solution. After 30 min of incubation at room temperature, the absorbance of the samples was measured at 660 nm; the same reaction solution without S3P was used as the blank control. The *K*_*m*_ values for substrates, *K*_*i*_ value and IC_50_ value for glyphosate were calculated as described by Tian *et al.*^12^.

### Generation of transgenic rice

To evaluate the potential application of the isolated *aroA*_*J. sp*_ gene in developing glyphosate tolerant crops, a *japonica* rice variety, zhonghua11 (ZH11) and a *indica* rice variety, minghui86 (MH86), were selected as the plant materials for genetic transforamtion. The chloroplast targeting signal peptide (CTP) coding sequence is from *Arabidopsis thaliana* and contains 228 nucleotides (Genebank:AAB72287.1). The nucleotide sequence of the *aroA*_*J. sp*_ gene and CTP were codon-optimized according to the codon bias in rice and was chemically synthesized to produce *ctp-aroA*_*J. sp*_ fusion gene (Takara, Japan). The *hpt* expression cassette in the binary vector pCAMBIA1300 (provided by the Center for the Application of Molecular Biology in International Agriculture, Australia) was removed by using *Xho*I and *EcoR*I restriction sites, resulting in pU130 transformation vector. Maize Ubi promoter and *ctp-aroA*_*J. sp*_ fusion gene were subcloned into pU130 using mutiple clone sites, and pU130-*aroA*_*J. sp*_ (*Ubi-*1: *aroA*_*J. sp*_: *35S polyA*) was obtained. The pU130-*aroA*_*J. sp*_ was introduced into *A. tumefaciens* EHA105 by electroporation, and by following the callus culture and tansformation procedures as described by Hiei *et al.*^44^ the resistant calli were obtained from the selection medium containing 200 mg/L glyphosate.

### Molecular analysis of transgenic rice

To confirm the correct integration of the *aroA*_*J. sp*_ gene into the rice genome, the genome DNA from young leaves of rice plants was extracted by CTAB method^45^ and used as the template for PCR to amplify an 815 bp fragment of *aroA*_*J. sp*_ with Primer 5 (5′-TTCCTTAAAGCGAAAACCCC-3′) and Primer 6 (5′-AGGAGGGCGGACACGAACTG-3′). Expression of *aroA*_*J. sp*_ gene in T_0_ transgenic rice was analyzed by RT-PCR. Total RNA from rice plants was extracted using the Trizol reagent (Transgen, China) according to the manufacturer’s instructions. For the first strand cDNA synthesis, 1 μg of the total RNA was used with M-MLV reverse transcriptase (Invitrogen, USA) after DNase I digestion (Invitrogen, USA). The endogenous *actin* gene was amplified with the primers *actin*-F (5′-GCCACACTGTCCCCATCTAT-3′) and *actin*-R (5′-GCGACCACCTTGATCTTCAT-3′) as an internal control. The *ctp-aroA*_*J. sp*_ fragment was amplified with primer 7 (5′-GCATGCTCTCCCCGGATTGG-3′) and primer 8 (5′-GAGCTCCTATCAGCCCTCGGA-3′), resulting in 1.3 kp amplified DNA product. For Southern blot analysis, 10 μg of genomic DNA digested with *Hind*III or *Sac* I was electrophoresed on 0.8% agarose gel and transferred onto a Hybond^+^ nylon membrane (GE Healthcare UK Limited). The *aroA*_*J. sp*_ probe was DIG labeled using Primer 5 (5′-TTCCTTAAAGCGAAAACCCC-3′) and Primer 6 (5′-AGGAGGGCGGACACGAACTG-3′). Hybridization and detection steps were performed according to the manufacturer’s instruction (Roche, Mannheim, Germany).

### Glyphosate tolerance assay of transgenic rice

Glyphosate-tolerance of transgenic rice plants was assayed by herbicide spraying. The T_0_ and T_2_ transgenic rice plants containing *aroA*_*J. sp*_ gene were planted in the weed controlled field and sprayed with either different amounts of glyphosate or 1% (vol/vol) solution of the herbicide Roundup which contains 41% isopropylamine salt of glyphosate (Monsanto, Malaysia). In addtion, T_4_ generation seedlings of ME1 line were also transplanted into a weedy field without weed control to investigate the potential to be used in no-till system. In the weedy field, there were mainly Gramineae weeds (*Echinochloa Crusgalli* (L.) Beauv), Cyperaceae weeds (*Cyperus difformis* L. & *Cyperus fuscus* L.), Pontederiaceae weeds (*Monochoria vaginalis* (Burm.f.) Presl ex Kunth) and Scrophulariaceae weeds (*Lindernia procumbens* (Krock.) Borbas) in this research.

## Additional Information

**How to cite this article**: Yi, S.- *et al.* A Novel Naturally Occurring Class I 5-Enolpyruvylshikimate-3-Phosphate Synthase from *Janibacter* sp. Confers High Glyphosate Tolerance to Rice. *Sci. Rep.* **6**, 19104; doi: 10.1038/srep19104 (2016).

## Supplementary Material

Supplementary Information

## Figures and Tables

**Table 1:** Kinetic properties of aroA_*J. sp*_ and aroA_*E. coli*_^a^.

Enzyme	Sp act (nKat/mg)	*K*_*m*_(PEP)^b^ (μM)	*K*_*m*_(S3P)^b^ (μM)	*K*_*i*_ c (μM)	IC_50_^d^ (mM)	*K*_*i*_/*K*_*m*_(PEP)
AroA_*J. sp*_	27.9 ± 3	30 ± 4	83 ± 11	373 ± 50	3.6 ± 0.2	12.4
AroA_*E. coli*_	15.6 ± 4	60 ± 7	115 ± 10	0.9 ± 0.09	0.04 ± 0.01	0.015

^a^The results are the averages of two sets of experiments conducted in triplicate.

^b^The PEP or S3P concentration was set at 0.05, 0.067, 0.1, 0.2, 0.5, and 1 mM, while the concentration of the other one was fixed at 1.0 mM.

^c^Competitive inhibition by glyphosate with respect to PEP was demonstrated by lines converging on the x axes of Lineweaver-Burk plots. The PEP concentration was set at 0.067, 0.1, 0.2, and 0.5 mM, respectively, while the glyphosate concentration was 0, 5, 10, 20, and 50 μM in determining the inhibition of aroA_*E. coli*_; and the glyphosate concentration was 0, 0.1, 0.5, 1, 2 and 5 mM in determining the inhibition of aroA_*J. sp*_. S3P concentration was fixed at 1 mM.

^d^The glyphosate concentration causing 50% inhibition of enzyme activity, which was determined by fitting the data to the equation: V = V min + (V max −V min)/(1 + ([I]/IC 50)^n^), and V was determined at 1 mM PEP and 1 mM S3P with the glyphosate concentration ranging from 0.0001 mM to 100 mM.

**Table 2:** Statistical analysis of transformation efficiency in *japonica* rice, ZH11.

Replicates	Number of calli inoculated	Glyphosate-resistant calli	Resistant calli formation rate (%)	Resistant calli that regenerated plants	PCR positive independent lines	Single-copy independent lines
1	148	84	56.8	16	16	6
2	124	67	54.0	8	8	2
3	165	75	45.5	13	13	6

**Table 3:** Comparison of agronomic traits between transgenic homozygous lines and transgenic-negative lines under field conditions^a^.

	Plant height (cm)	Panicle per plant	Panicle length (cm)	Seed-set rate	Weight per 1,000 grains (g)	Yield/plant (g)
ME1-P	110.50 ± 3.36	12.30 ± 2.00	25.75 ± 0.39	0.73 ± 0.02	23.36 ± 1.13	32.11 ± 2.70
ME1-N	112.00 ± 1.30	12.70 ± 0.44	27.33 ± 1.33	0.70 ± 0.03	22.76 ± 1.10	32.20 ± 1.74
ME2-P	107.05 ± 3.20	12.73 ± 1.76	24.80 ± 0.44*	0.71 ± 0.06	23.86 ± 1.13	30.27 ± 9.22
ME2-N	112.87 ± 2.66	14.63 ± 1.14	25.94 ± 0.40	0.74 ± 0.01	24.29 ± 1.01	38.72 ± 2.54

ME1-P and ME2-P represent homozygous transgenic lines of ME1 and ME2, while ME1-N and ME2-N are their corresponding negative transgenic lines.

^a^The parameters were given as means (±standard deviation) for data collected from 10 plants in triplicate for each plant type.

^*^Means significantly different from the control (P < 0.05).
