# Supplementary material for: A Novel Naturally Occurring Class I 5-Enolpyruvylshikimate-3-Phosphate Synthase from Janibacter sp. Confers High Glyphosate Tolerance to Rice
Source: Sci Rep. 2016 Jan 12;6:19104. doi: 10.1038/srep19104 (PMC4709569; doi:10.1038/srep19104)
Supplement: Supplementary Information [file srep19104-s1.doc]

**A Novel Naturally Occurring Class I 5-Enolpyruvylshikimate-3-Phosphate Synthase from *Janibacter* sp.** **Confers High Glyphosate Tolerance to Rice**

Shu-yuan Yi^1^ ^&^, Ying Cui^2&^, Yan Zhao^1^, Zi-duo Liu^1^, Yong-jun Lin^2^, Fei Zhou^2*^

^1^State Key Laboratory of Agricultural Microbiology, College of Life Science and Technology, Huazhong Agricultural University, Wuhan 430070, China

^2^National Key Laboratory of Crop Genetic Improvement and National Centre of Plant Gene Research, Huazhong Agricultural University, Wuhan 430070, China

^&^ These authors contributed equally to the work.

* Corresponding author:

E-mail address: zhoufei@mail.hzau.edu.cn;

Tel: 86-15207187316; Fax: 86-27-87280550

**Supplementary figure S1**


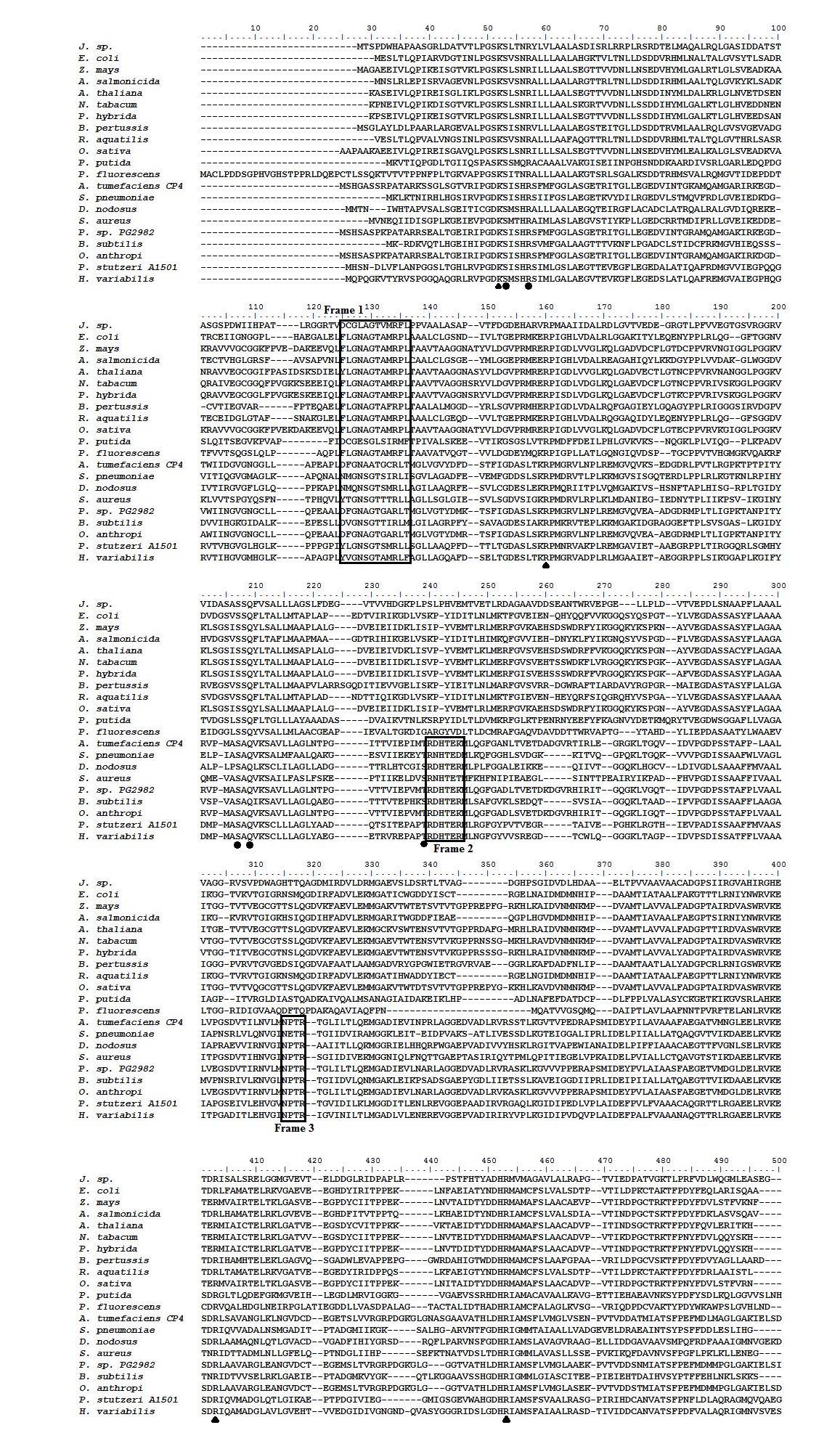


Figure S1. Multiple alignments of amino acid sequences from aroA*_J. sp_* with representative class I and class II AroA enzymes using ClustalW program. Triangles, residues critical for PEP binding; and circles, residues critical for S3P binding. The two regions involved in glyphosate resistance in class II AroA enzymes are boxed in Frames 2 and 3. The motif important for interaction with PEP conserved in class I AroA enzymes is boxed in Frame 1.

**Supplementary figure S2**


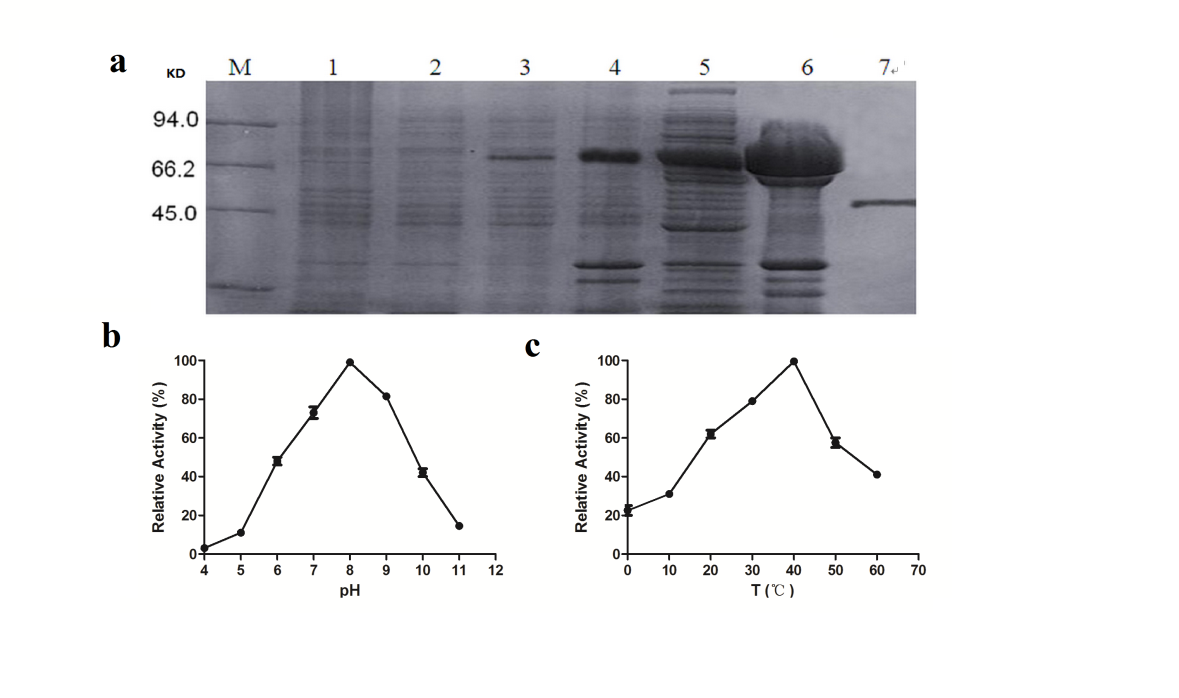


Figure S2. a, A 12% SDS-PAGE analysis of the purified aroA enzyme by recombined expression. Lane M, standard protein marker; lane 1, IPTG-induced cell lysate of *E. coli* BL21 (DE3) harboring pGEX-6p-1; lane 2, un-induced cell lysate of *E. coli* BL21 (DE3) harboring pGEX-6p-1; lane 3, un-induced cell lysate of *E. coli* BL21 (DE3) harboring pGEX-6p-1- *aroA_J. sp_*; lane 4, un-induced cell pellet of *E. coli* BL21 (DE3) harboring pGEX-6p-1- *aroA_J. sp_*; lane 5, IPTG-induced cell lysate of E. coli BL21 (DE3) harboring pGEX-6p-1- *aroA_J. sp_*; lane 6, IPTG-induced cell pellet *E. coli* BL21 (DE3) harboring pGEX-6p-1- *aroA_J. sp_*; lane 7, purified aroA_J. sp_ enzyme (5µg). b, the optimum pH. Enzyme activity was measured at pH ranging from pH 4-11. c, the optimum temperature. Enzyme activity was measured at temperatures ranging from 0 °C to 60 °C.
